# Multitask Deep Neural Network for the Fully Automatic Measurement of the Angle of Progression

**DOI:** 10.1155/2022/5192338

**Published:** 2022-09-02

**Authors:** Yaosheng Lu, Dengjiang Zhi, Minghong Zhou, Fan Lai, Gaowen Chen, Zhanhong Ou, Rongdan Zeng, Shun Long, Ruiyu Qiu, Mengqiang Zhou, Xiaosong Jiang, Huijin Wang, Jieyun Bai

**Affiliations:** ^1^College of Information Science and Technology, Jinan University, Guangzhou, Guangdong, China; ^2^Department of Obstetrics and Gynecology, Chengdu Women's and Children's Central Hospital, School of Medicine, University of Electronic Science and Technology of China, Chengdu 611731, China; ^3^Obstetrics and Gynecology Center, Zhujiang Hospital, Southern Medical University, Guangzhou, Guangdong, China

## Abstract

The angle of progression (AoP) for assessing fetal head (FH) descent during labor is measured from the standard plane of transperineal ultrasound images as the angle between a line through the long axis of pubic symphysis (PS) and a second line from the right end of PS tangentially to the contour of the FH. This paper presents a multitask network with a shared feature encoder and three task-special decoders for standard plane recognition (Task1), image segmentation (Task2) of PS and FH, and endpoint detection (Task3) of PS. Based on the segmented FH and two endpoints of PS from standard plane images, we determined the right FH tangent point that passes through the right endpoint of PS and then computed the AoP using the above three points. In this paper, the efficient channel attention unit is introduced into the shared feature encoder for improving the robustness of layer region encoding, while an attention fusion module is used to promote cross-branch interaction between the encoder for Task2 and that for Task3, and a shape-constrained loss function is designed for enhancing the robustness to noise based on the convex shape-prior. We use Pearson's correlation coefficient and the Bland–Altman graph to assess the degree of agreement. The dataset includes 1964 images, where 919 images are nonstandard planes, and the other 1045 images are standard planes including PS and FH. We achieve a classification accuracy of 92.26%, and for the AoP calculation, an absolute mean (STD) value of the difference in AoP (∆AoP) is 3.898° (3.192°), the Pearson's correlation coefficient between manual and automated AoP was 0.964 and the Bland-Altman plot demonstrates they were statistically significant (*P* < 0.05). In conclusion, our approach can achieve a fully automatic measurement of AoP with good efficiency and may help labor progress in the future.

## 1. Introduction

Cesarean section is an important procedure for both the mother and the fetus in certain medical conditions [1–4]. However, an unnecessary cesarean section can lead to higher medical risks for both mothers and infants [3]. Therefore, proper maternal and fetal monitoring during labor is very important because this is the only way to assess the progress of labor and identify deviations from the normal. The clinical variable of the head movement of the fetal is used to inform decision-making regarding mode of delivery during active pushing [5]. In clinical practice, digital examination is a fundamental method for monitoring the descent of the fetal head (FH), but the method is known to have limited accuracy [6–8] and repeated screening may lead to vaginal bacteria entering the cervix and the uterus and causing harm to the newborn [9].

**Figure 1:**
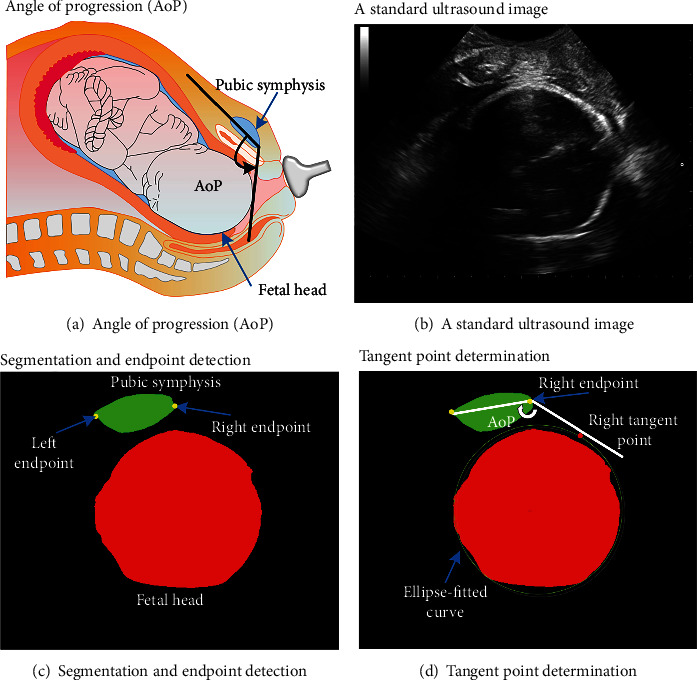
A schematic overview of automatic measurement of AoP based on ultrasound images. (a) Schematic diagram of AoP that is measured transperineally as the angle between a line through the long axis of the PS and a second line from the inferior end of the PS tangentially to the contour of the FH. (b) A standard ultrasound image including the PS and the FH. (c) Regions of the PS (green) and the FH (red) are segmented and the endpoints of the PS are determined from the standard ultrasound image. (d) Based on the segmented FH, the contour of the FH is determined by fitting an ellipse equation. Then, the right tangent connected to the right endpoint of the PS is retained to calculate AoP.

Recently, several studies have indicated that ultrasound measurements are more accurate and repeatable than digital examination [10–17], and angle of progression (AoP) is found to be the most reproducible ultrasound parameter when examining FH descent [18–21]. AoP is measured transperineally as the angle between a line through the long axis of pubic symphysis (PS) and a second line from the inferior end of the PS tangentially to the contour of the fetal skull (Figure 1(a). Barbera et al. are the first to use transperineal ultrasound (TPU) to manually measure AoP [22]. And AoP has been found to be correlated to the ischial spines in different studies [23–25]. Tutschek et al. found that the zero station of FH corresponds to an AoP of 116 degrees [26]. Arthuis et al. researched computed tomographic (CT) images and found that the ischial spines are associated with an AoP of 110 degrees [24], while Bamberg et al. related an AoP of 120 degrees to the ischial spines obtained with magnetic resonance imaging (MRI) [25]. Moreover, a comparison between the MRI and CT methods showed a mean difference of only 1.4 degrees [27]. A similar, feasible, and highly reproducible method was also used to examine FH descent in breech-presenting fetuses [28]. The use of TPU to simultaneously assess the FH descent would be desirable; however, it is technically challenging for nonexperienced operators to diagnose the FH descent using TPU [29].

Recently, Obstetrics and Gynecology have introduced new techniques to provide fast and automatic identification and measurement of normal and abnormal ultrasound examination results [30, 31]. Conversano et al. reported a real-time tracking algorithm for noninvasive and automatic monitoring of AoP during the second stage of labor [32, 33]. In the process of AoP measurement, the initial standard plane including PS and FH was manually identified according to targets within the TPU image and the gray level values of their pixels. The two substructures (i.e., PS and FH) in the initial image were automatically segmented and identified as the two patterns to be searched within the subsequent images by maximization of similarity or cross-correlation coefficients [32]. The axis and distal end of PS were segmented in subsequent images, and displacements from the previous position were also calculated. Simultaneously, the pattern location of FH was employed to initialize the automatic edge outlining from subsequent images and to calculate the displacement of the rightmost point of the FH from the previous position. Finally, the coordinates and displacements of the FH for each frame were determined regarding the reference system associated with the PS distal end to calculate AoP. Different from the above method developed by Conversano et al., a deep learning-based approach was first developed and tested preliminarily on a small dataset by Zhou et al. [33]. Firstly, the landmark of PS endpoints was located and areas of PS and FH were segmented by a deep learning network. Secondly, the central axis of PS was obtained with the two endpoints, which are physical points that are used for the determination of AoP. Thirdly, the tangle of FH was determined as it passed through the lower endpoint of PS. Finally, AoP was calculated from the central axis and the tangent. All of these methods are based on the standard planes of the TPU images. Therefore, an end-to-end method for fully automatic measurement of AoP should be further developed.

This paper presents a fully automatic measurement framework of AoP for the multitask process that includes standard plane recognition (Figure 1(b), Task1), image segmentation (Figure 1(c), Task2) of PS and FH, endpoint detection (Figure 1(c), Task3), and AoP calculation (Figure 1(d), AoP calculation). In the framework, a multitask Unet (MT-Unet) with a shared feature encoder and three task-special decoders is proposed for the above three tasks. More specifically, the efficient channel attention (ECA) unit in the shared encoder, attention fusion module (AFM) between decoders, and a designed shape-constrained loss function (SLF) are used to improve the performance of our MT-Unet. Based on the segmented FH of Task2 and the detected endpoints, the tangent point of FH is determined, and thereby, AoP is calculated. In brief, our main contributions include the following:

1. A two-stage measurement framework of AoP: MT-Unet is used for standard plane recognition, image segmentation, and endpoint detection in the first stage, while tangent point determination and AoP calculation are conducted in the second stage
2. Based on the multitask process for AoP calculation from TPU images, an MT-Unet is designed for this application. Various modules (including ECA, AFM, and SLF) are used for improving its performance
3. Our method outperforms existing deep learning methods for automatic AoP measurement

The remainder of this paper is structured as follows.  Section 2 mainly explains our method, experimental details and dataset, etc.  Section 3 presents the experimental results, and Section 4 provides some discussion, before some concluding remarks in Section 5.

## 2. Materials and Methods

**Figure 2:**
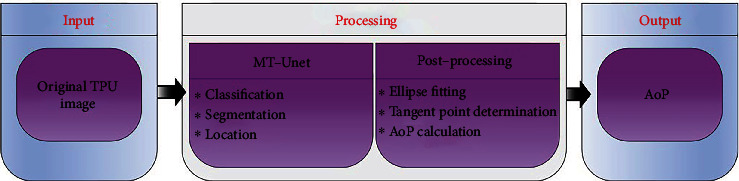
Illustration diagram of the proposed algorithm for automatic measurement of angle of progression based on TPU images.

An outline of the proposed automatic AoP measurement algorithm is shown in Figure 2. Firstly, standard ultrasound images of the original TPU images (i.e., input) are selected, target areas are segmented, and two endpoints are determined based on the proposed MT-Unet. Secondly, the contour of the region of the FH is fitted with an ellipse equation, and then, the right tangent point connected to the right endpoint is determined. Finally, AoP (i.e., output) is calculated as the angle between a line through two endpoints and a second line through the right endpoint and the tangent point. In short, the automatic AoP measurement algorithm mainly includes the MT-Unet and postprocessing parts.

### 2.1. MT-Unet

Taking into account the characteristics of different tags and tasks of the same input image data, we propose a network with one shared encoder and three task-specific decoders inspired by Zhou et al. [34]. Three main modifications are applied to the MT-Unet for accuracy improvement. The ECA module is used to capture local cross-channel interaction in the shared encoder, the AFM module is used to capture cross-branch interaction among the task-specific decoders, and SLF is designed based on the prior convex shape.

#### 2.1.1. Network Architecture

**Figure 3:**
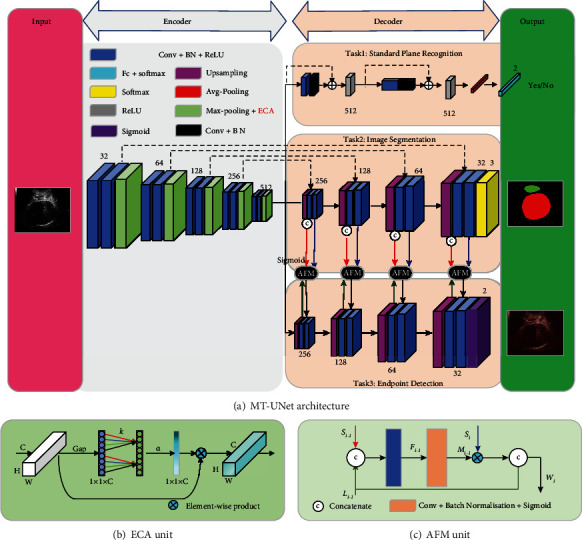
The proposed MT-Unet network structure. (a) The MT-Net architecture is composed of an encoder and three decoder tasks (i.e., Task1: image classification; Task2: image segmentation; Task3: position location). (b) Diagram of the ECA unit. (c) Diagram of the AFM unit.

The MT-Unet architecture is illustrated in Figure 3 which shows one shared encoder and three decoders (Figure 3(a)).

The encoder is composed of five blocks, each of which contains two convolutional layers and a downsampling layer. The convolution layer includes a convolutional operation (Conv) with a kernel size of 3 × 3, a batch normalization layer (BN), and a rectified linear unit (ReLU). The downsampling layer consists of a maximum pooling (Max-pooling) operation with a kernel size of 2 × 2 and an ECA unit.

Three task-special decoders are designed for standard plane recognition (i.e., Task1), image segmentation (i.e., Task2), and endpoint detection (i.e., Task3). The decoder for Task1 consists of two ResBlocks, and each of them contains a convolutional layer followed by Conv, BN, an operation of shortcut connection, and ReLU. The final output is followed by an average pooling (Avg-pooling) operation, a fully connected (FC) layer, and a Softmax. The decoder for Task2 is made up of four upsampling blocks and a Softmax layer. Each up-sampling block contains an upsampling layer and two convolutional layers. Inspired by Unet [35], we introduced skip connections into Task2. Similarly, Task3 has four upsampling blocks as well, but uses Sigmoid as its activation function. Several AFM units are used to fuse features between Task3 and Task2.

#### 2.1.2. ECA Unit

The ECA module is used to capture local cross-channel interaction considering each channel and its *k* neighbors [36]. Given the aggregated feature (*C* × *H* × *W*) using channel-wise global average pooling (GAP), it generates channel weights by performing a fast 1D convolution of size *k* followed by a Sigmoid function (*α*) (Figure 3(b)).

The kernel size *k* represents the coverage of the local cross-channel interaction, i.e., the number of neighbors participating in channel attention prediction of each channel. *k* is adaptively determined via a function of channel dimension *C*.

(1) $$\begin{array}{c} k=\varphi \left(C\right)={\left|\frac{\log_{2}\left(C\right)+b}{\gamma }\right|}_{\text{odd}}, \end{array}$$

where |*t*|_odd_ represents the nearest odd number of *t*. In the present study, we set *γ* and *b* as 2 and 1, respectively.

#### 2.1.3. AFM Unit

The AFM is designed to allow the network to learn task-related features. These features include the shared features (red arrow, *S*_*i*−1_) of the Task2 branch and task-specific features (green arrow, *L*_*i*−1_) from the previous layer. The concatenation of task-related features (i.e., *S*_*i*−1_ and *L*_*i*−1_) is the input of the AFM. This input followed by a 1 × 1 convolutional layer (blue block) is used to generate a feature map (*F*_*i*−1_), and *F*_*i*−1_ is input into a block (including a 1 × 1 Conv, BN, and a Sigmoid activation operation) (orange block) to generate an attention mask (*M*_*i*−1_) with intensity within [0, 1]. A weighted feature map (black arrow, *W*_*i*_) of the current layer is generated with the concatenation of *L*_*i*−1_ and the product of element-wise multiplication of *M*_*i*−1_ and the shared features (blue arrow, *S*_*i*_) of the current layer. *W*_*i*_ is merged into the Task3 branch by channel-dimensional concatenation.

#### 2.1.4. Loss and Optimization

The MT-Unet is trained in a two-stage manner. In the first stage, the network with the task-specific decoders (i.e., Task2 and Task3) is trained with a linear combination of a set of loss functions as a pretrained model, which is a model created by someone else to solve a similar problem. Here, Dice loss and shape-constrained loss are used in Task2 and mean squared error loss [37] in Task3. In the second stage, the pretrained model with the shared encoder and the Task1 decoder is trained with the cross-entropy loss. It is worth noting that the parameters of the shared encoder are loaded from the pretrained model of the first stage.

The cross-entropy loss function (*L*_CE_) in Task1 for binary classification is used [38]. It measures the difference between two probability distributions of the predicted value and the ground truth label and is equivalent to the negative log-likelihood loss as follows:

(2) $$\begin{array}{c} L_{\text{CE}}\left(y,\hat{y}\;\right)=-y\log\left(\hat{y}\right)-\left(1-y\right)\log\left(1-\hat{y}\right), \end{array}$$

where *y* and $\hat{y}$ refer to the ground truth label and the predicted value, respectively.

The loss consists of three components: Dice loss (*L*_D_) for image segmentation of Task2 [39], shape-constrained loss (*L*_SC_) for convex-shape segmentation of Task2 [40], and mean squared error loss (*L*_MSE_) for endpoint location of Task3 [41]. The total loss is given by

(3) $$\begin{array}{c} L_{\text{total}}=w_{1}\left(\theta_{1}L_{\text{D}}+\theta_{2}L_{\text{SC}}\right)+\left(1-w_{1}\right)L_{\text{MSE}}, \end{array}$$

where *θ*_1_ and *θ*_2_ are scaling factors determined via the weight uncertainty method [42]. *w*_1_ (0.5) is obtained via hyperparameter analysis.

Dice loss (*L*_D_):

(4) $$\begin{array}{c} L_{\text{D}}=1-\frac{2\sum_{i=1}^{N}\sum_{j=1}^{C}y_{i,j}p_{i,j}}{\sum_{i=1}^{N}\sum_{j=1}^{C}\left(y_{i,j}+p_{i,j}\right)}, \end{array}$$

where *y* is the ground truth map, *p* is its corresponding predicted map, *N* is the number of pixels, and *C* is the number of classes (excluding the background).

Shape-constrained loss (*L*_SC_):

(5) $$\begin{array}{c} L_{\text{SC}}=\underset{i\in I}{\sum }\underset{p,q\in I}{\sum }\underset{r\in l_{pq}}{\sum }B_{pqr}^{i}\left|y_{ip}-p_{ip}\right.\left\|y_{iq}-p_{iq}\right\|\left.p_{ip}+p_{iq}-2p_{ir}\right|, \end{array}$$

where $B_{pqr}^{i}=\left\{\begin{array}{c} 1,\text{ if }y_{ip}=y_{ip}=y_{ir}=1\\ 0,\text{ otherwise} \end{array}\right.$, where *p*, *q*, and *r* are three points. (*p*, *q*) is a point pair inside the segmented region, and *r* is on the line (*l*_*pq*_) that is bounded by *p* and *q*, *y*_*ip*_ and *y*_*iq*_ are the ground truth labels, while *p*_*ip*_, *p*_*iq*_, and *p*_*ir*_ are the predicted values. *L*_SC_ can be activated when *p*, *q*, and *r* have the same label (i.e., *B*_*pqr*_^*i*^=1) [40].

Mean squared error loss (*L*_MSE_):

(6) $$\begin{array}{c} L_{\text{MSE}}=\overset{n}{\underset{k=1}{\sum }}\delta_{k}\underset{\text{i},\text{j}}{\sum }{\left\|H_{k}\left(i,j\right)-{\overset{\wedge }{H}}_{k}\left(i,j\right)\right\|}^{2}, \end{array}$$

where *n* = 3 is the number of points, *δ*_*k*_ is the loss weight, and *H*_*k*_ and ${\hat{H}}_{k}$ represent the predicted heatmaps [33] and the ground-truth heatmaps, respectively. Here, *δ*_1_, *δ*_2_, and *δ*_3_ are set to be 1.0, 0.8, and 0.6 according to the importance of each heatmap, respectively.

### 2.2. Postprocessing

The output of MT-Unet includes image category, segmented regions, and coordinates of the two endpoints (i.e., right and left endpoints) of the PS. In order to measure AoP, the line from the right endpoint of the PS tangentially to the contour of the FH should be determined. Therefore, the contour of the FH is determined by fitting an ellipse equation through the least square method [43, 44], and then, the right tangent connected to the right endpoint of the PS is retained to calculate AoP (see Appendix S1 for details).

### 2.3. Experimental Setup

#### 2.3.1. Dataset

Our dataset consists of 1964 TPU images of 104 volunteers during labor. These images with a resolution of 800 × 652 in BMP format were retrospectively collected from the Zhujiang Hospital of Southern Medical University between 2020 and 2021. TPU examinations were performed in standard B-mode ultrasound using Esaote ultrasound systems. The dataset was divided into two parts (i.e., one includes 1045 standard plane images, while the other includes 919 nonstandard plane images) to generate the image-level labels for image classification. For these standard plane images, three types of pixel-level labels were annotated by a team of 4 expert sonographers and then manually validated. The first type of pixel-level labels is regions of FH and PS for image segmentation, the second type is two endpoints of PS for key point positioning, and the third type is AoP.

#### 2.3.2. Preprocessing

This image dataset was randomly divided into training, validation, and testing sets in a ratio of 5 : 2 : 3. Since the image dataset includes standard plane set (1045 standard plane images) and nonstandard plane set (919 nonstandard plane images), the two sets are also split into training, validation, and testing sets in a ratio of 5 : 2 : 3. Since each patient had multiple images, the data was split so that all images from a patient were only in one of the training, validation, and testing sets. Furthermore, we adopted a two-stage training strategy to obtain our MT-Unet. Both standard and nonstandard plane images were used for Task1 at the second stage, but only standard plane images were used for Task2 and Task3 at the first stage. Random rotation (−30°, 30°) and random scaling were used for data augmentation during training; the input images were resized to a size 416 × 384 and normalized to [−1, 1].

#### 2.3.3. Training Settings

The adaptive moment estimation optimizer [45] was used for optimization. We used the step learning rate scheduler [46] (StepLR) with a step size of 20. The learning rate scheduler was adopted to decrease the learning rate from its initial value (0.0001) by a factor gamma (0.1). The network weights were initialized via the Kaiming initialization [47] and trained for 200 epochs with a batch size of 2. All experiments have been carried out based on PyTorch [48] and run on a Nvidia Titan V GPU.

#### 2.3.4. Evaluation Metrics

Different performance metrics have been adopted for image classification, image segmentation, endpoint detection, and AoP calculation.

For the image classification task (Task1), we employed accuracy (*Acc*), precision (*Pre*), Sensitivity (*Sen*), and Specificity (*Spe*).

(7) $$\begin{array}{c} \text{Acc}=\frac{\text{TP}+\text{TN}}{\text{TP}+\text{FP}+\text{TN}+\text{FN}},\\ \text{Pre}=\frac{\text{TP}}{\text{TP}+\text{FP}},\\ \text{Sen}=\frac{\text{TP}}{\text{TP}+\text{FN}},\\ \text{Spe}=\frac{\text{TN}}{\text{FP}+\text{TN}}, \end{array}$$

where TP, FP, FN, and TN denote true positive, false positive, false negative, and true negative.

For the image segmentation task (Task2), we used *Acc*, Dice scores of the PS (*Dice_PS*), the FH (*Dice_FH*), and both targets (*Dice_ALL*).

(8) $$\begin{array}{c} \text{Dice}=\frac{2\text{TP}}{2\text{TP}+\text{FP}+\text{FN}}. \end{array}$$

For the endpoint detection task (Task3), we firstly located the two pubic symphysis endpoints by regressing Gaussian heatmaps [49] and then used the Euclidean distance (*Dist*) between the predicted endpoint coordinate (*x*_*p*_, *y*_*p*_) and the corresponding ground-truth coordinate (*x*_*t*_, *y*_*t*_). Two distances in *Dist_L* and *Dist_R* were introduced to evaluate the performance of Task3 for the left and right endpoints.

(9) $$\begin{array}{c} \text{Dist}={\left[{\left(x_{t}-x_{p}\right)}^{2}+{\left(y_{t}-y_{p}\right)}^{2}\right]}^{0.5}. \end{array}$$

For AoP calculation, we evaluated the angle (APT) between the predicted line ($\overset{⟶}{L_{p}}$) through the predicted left endpoint (*x*_*pl*_, *y*_*pl*_) and the predicted right endpoint (*x*_*pr*_, *y*_*pr*_) and its the corresponding ground-truth line ($\overset{⟶}{L_{t}}$) through two endpoints (i.e. (*x*_*tl*_, *y*_*tl*_) and (*x*_*tr*_, *y*_*tr*_)).

(10) $$\begin{array}{c} \overset{⟶}{L_{p}}=\left[x_{pr}-x_{pl},y_{pr}-y_{pl}\right]\overset{⟶}{L_{t}}=\left[x_{tl}-x_{tr},y_{tl}-y_{tr}\right],\\ \text{APT}=\cos^{-1}\frac{\overset{⟶}{L_{p}}∙\overset{⟶}{L_{t}}}{\left|\overset{⟶}{L_{p}}\right|\left|\overset{⟶}{L_{t}}\right|}. \end{array}$$

In addition, the absolute value of the difference in AoP (∆AoP) between the predicted AoP (AoP_*p*_) and the clinically acquired one (AoP_*t*_) is an important evaluation metric.

(11) $$\begin{array}{c} ∆\text{AoP}=\left|{\text{AoP}}_{p}-{\text{AoP}}_{t}\right|. \end{array}$$

## 3. Results

### 3.1. Fully Automated Measurement of AoP

Experimental results suggest that our image classification has achieved an *Acc* of 92.26% in terms of distinguishing whether an image is a standard plane image that contains PS and FH.

**Figure 4:**
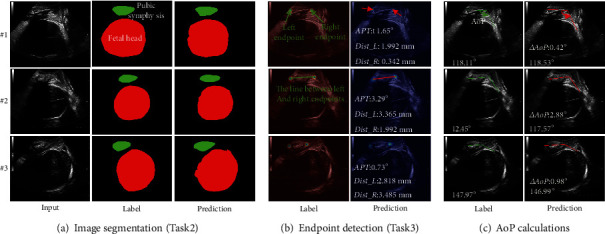
Examples of MT-Unet on the PS-FH. (a) Prediction examples (i.e., #1, #2, and #3) of the MT-Unet segmentation task. (b) Prediction examples of the MT-Unet endpoint detection task. (c) Prediction examples of AoP calculation. Note: *Dist_L/Dist_R* denotes the Euclidean distances between the predicted left/right endpoints and the true left/right endpoints. APT denotes the angle between the true line and the predicted line of two endpoints. ∆AoP denotes the absolute value of AoP difference between the true AoP and the predicted one.

From standard plane images, image segmentation (Task2), endpoint location (Task3), and AoP calculations have been conducted, and the overall performance of the whole test cases of our method on Acc, Dice_ALL, Dice_PS, Dice_FH, Dist_L, Dist_R, APT, and ∆AoP is 98.9%, 92.0%, 84.8%, 93.2%, 6.303 mm, 5.789 mm, 6.500°(±5.121°), and 3.898°(±3.192°), and examples of the results are shown in Figure 4 (see Appendix S2 for details).

For Task2, ground truth target masks and segmented results using our MT-Unet are compared in Figure 4(a). The ranges of the Dice score for the PS (*Dice_PS*), the FH (*Dice_FH*), and the two targets (*Dice_ALL*) are 89.0%-93.4%, 94.7%-98.3%, and 94.2%-97.3%.

For Task3, the predicted coordinate positions of the two endpoints of the PS are compared to the annotated ones (Figure 4(b)). For #1, #2, and #3, the distance differences between the left endpoints (*Dist_L*) are 1.992 mm, 3.365 mm, and 2.818 mm, whereas the distance differences between the right endpoints (*Dist_R*) are 0.342 mm, 1.99 mm, and 3.485 mm. In addition, APT are 1.65°, 3.29°, and 0.73° for #1, #2, and #3.

For the AoP calculation task (Figure 4(c)), the calculated AoP is compared to the labeled one and the differences (∆AoP) are less than 3°. The predicted/labelled AoP is 118.53°/118.11° for #1, 117.57°/120.45° for #2, and 146.99°/147.97° for #3.

### 3.2. Comparative Experiment

To investigate the effectiveness of key components in our MT-Unet, we conducted a series of comparative studies. We compare our Task1 with Vgg16 [50] and Resnet50 [51] for standard ultrasound images' identification with *Acc*, *Pre*, *Sen*, and *Spe*. Furthermore, we removed ECA, AFM, and SLF from the MT-Unet to form the MT-Unet_A and compared it against two independent Unet (Unet) used for segmentation and location to investigate the effectiveness of the multitask network. Based on the MT-Unet, we removed ECA and SLF to form MT-Unet_B, removed AFM and SLF to form MT-Unet_C, and removed SLF to form MT-Unet_D. Finally, we evaluate the performance in *Acc*, *Dice_ALL*, *Dice_PS*, *Dice_FH*, *Dist_L*, *Dist_R*, *APT*, and ∆AoP to investigate the effectiveness of the key components of our framework.

#### 3.2.1. Performance of Standard Plane Recognition (Task1)

The performance of our MT-Unet and its variants for standard plane selection (Task1) are listed in Table 1. Acc, Pre, Sen, and Spe of our method reached 92.26%, 90.42%, 86.57%, and 94.72%. Our proposed method outperformed Vgg16 [50], Resnet50 [51], and ConvNet [52] by approximately 2% in Acc, Pre, and Spe, but did not show a noticeable improvement in Sen. And our network is slightly better than Swin_transformer [53] in terms of Acc and Spe.

#### 3.2.2. Performance of Multitask Learning Network

**Figure 5:**
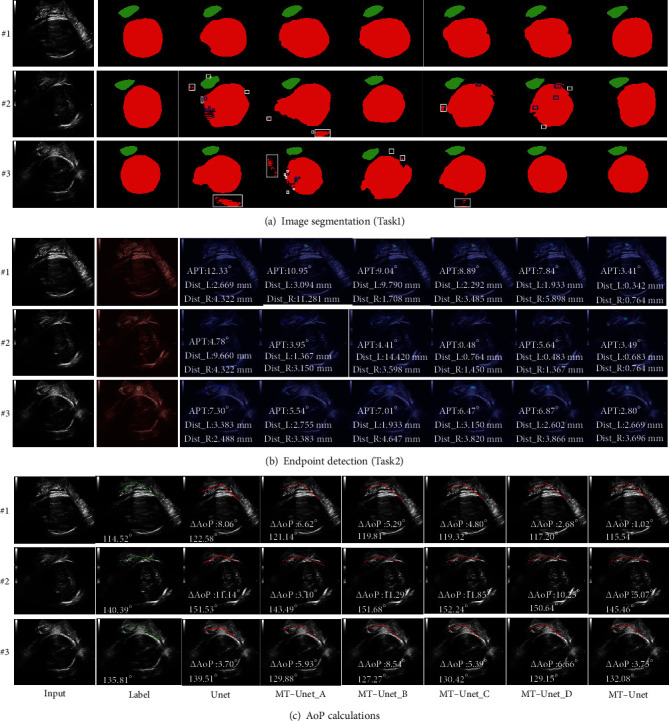
Summary of different methods. These methods include Unet, MT-Unet_A, MT-Unet_B, MT-Unet_C, MT-Unet_D, and MT-Unet. (a) Comparison of the prediction results of different segmentation methods. (b) Comparison of the prediction results of different location methods. (c) Comparison of AoP results of different methods. Compared with MT-Unet, MT-Unet_D is without SLF, MT-Unet_C is without SLF and AFM, MT-Unet_B is without SLF and ECA, MT-Unet_A is without SLF, AFM, and ECA. Note: Dist_L/Dist_R denotes the Euclidean distances between predicted left/right endpoints and true left/right endpoints. APT denotes the angle between the true line and the predicted line of two endpoints. ∆AoP denotes the absolute value of the AoP difference between the true AoP and the predicted one (complete and detailed results are provided in Appendix S3).

For Task2 (Figure 5(a)), the FH segmentation results for both methods (Unet and MT-Unet_A) are composed of multiple discrete regions that are distributed inside (marked with blue rectangles) and (or) outside (marked with white rectangles) of the labeled areas. Compare to ground truth target masks (#1 and #3), the main area segmented by MT-Unet_A was more accurate than that of Unet, but MT-Unet_A failed to provide good results in some complex situations (e.g., #2). Dice_FH/Dice_PS of MT-Unet_A vs. that of Unet is 92.2%/88.7% vs. 89.8%/83.6% for the case #1, 94.9%/90.6% vs. 96.4%/89.7% for the case #3, 88.9%/86.4% vs. 91.7%/79.3% for the case #2. The advantage of the multitask method is also reflected in the whole test dataset (MT-Unet_A/Unet): 97.6%/97.1% of Acc, 91.2%/90.1% of *Dice_ALL*, 85.3%/80.0% of *Dice_PS*, and 91.9%/91.5% of *Dice_FH*.

For Task3, the result of the endpoint location of the two methods is shown in Figure 5(b). The difference was quantified via Dist_L and Dist_R (i.e., the difference between the predicted and annotated coordinates). Dist_L/Dist_R achieved by Unet are 9.660/4.322 mm for #2, 3.383/2.488 mm for #3 and 2.669/4.322 mm for #1, whereas that of MT-Unet_A are 1.367/3.150 mm for #2, 2.755/3.383 mm for #3 and 3.094/11.281 mm for #1. Overall, the average Dist_L/Dist_R for MT-Unet_A on the whole test dataset is 8.131/9.109 mm, whereas that for Unet is 6.948/11.414 mm.

For AoP calculation, the effects of the two methods on AoP calculation can be evaluated via APT and ∆AoP. As shown in Figure 5(c), AoP obtained with MT-Unet_A/Unet is 121.14°/122.58° for #1, 143.49°/151.53° for #2, and 129.88°/139.51° for #3. Compared to the labelled AoPs (114.52° for #1, 140.39° for #2, and 135.81° for #3), ∆AoP of MT-Unet_A/that of Unet is 6.62°/8.06° for #1, 3.10°/11.14° for #2, and 5.93°/3.7° for #3. Changes in ∆AoP can be partly attributed to APT. APT of the MT-Unet_A/Unet is 10.95°/12.33° for #1, 3.95°/4.78° for #2, and 5.54°/7.30° for #3. The performance of the two methods on APT and ∆AoP demonstrates that the proposed multitasking method outperformed its task-oriented counterpart. The average APT/∆AoP on the whole test dataset of the MT-Unet_A is 9.047°(13.263°)/5.607°(5.725), whereas that of the Unet is 12.496°(18.756°)/6.646°(7.541°).

#### 3.2.3. Effectiveness of the ECA Module

The effects of the ECA module on the performance of MT-Unet were investigated through the following two pairs of experiments: MT-Unet_A vs. MT-Unet_C and MT-Unet_B vs. MT-Unet_D.

As is shown in Figure 5(a), when the ECA module is used in the multitask learning, the predicted discrete regions (white and blue rectangles) inside and outside the labeled area for the FH drastically shrunk and the resulting target areas were closer to the annotated regions (e.g., #1 and #3). These differences manifest as an increase in Dice scores. For case #2, Dice_PS (88.3% vs. 86.3%), Dice_FH (92.2% vs. 85.0%), and Dice_ALL (91.8% vs. 85.1%) of MT-Unet_D are larger than those of MT-Unet_B, and Dice_PS (88.2% vs. 86.4%), Dice_FH (92.4% vs. 88.9%), and Dice_ALL (92.0% vs. 88.7%) of MT-Unet_C are higher than that of MT-Unet_A. Similar results are obtained from the whole test dataset. Dice_ALL of MT-Unet_C vs. MT-Unet_A and MT-Unet_D vs. MT-Unet_B are 91.9% vs. 91.2% and 91.0% vs. 91.2% (details for Dice_PS and Dice_FH can be found in Table 2).

For Task3, Dist_L and (or) Dist_R were found lower in most cases (#1 and #2) when the ECA module has been used in the multitask method. For the case #2, Dist_L and Dist_R (MT-Unet_D vs. MT-Unet_B) are 0.483 mm vs. 14.420 mm and 1.367 mm vs. 3.598 mm. Dist_L and Dist_R (MT-Unet_C vs. MT-Unet_A) are 0.764 mm vs. 1.367 mm and 1.450 mm vs. 3.150 mm. However, Dist_L and (or) Dist_R were found to be higher in case #3. The performance of these methods on the whole test dataset demonstrated that the averages of Dist_L and Dist_R are reduced with the introduction of the ECA module. The average Dist_L is reduced from 9.105 mm to 6.638 mm, whereas the average Dist_R is reduced from 8.198 mm to 5.586 mm (MT-Unet_B vs. MT-Unet_D). Similar results were also obtained for MT-Unet_C vs. MT-Unet_A (Table 2).

For AoP calculation, when the ECA module is used in the multitask Unet, APT was reduced for #2(3.95°/0.48°) and #1(10.95°/8.89°) in the case of MT-Unet_A vs. MT-Unet_C, and it decreased for #3(7.01°/6.87°) and #1(9.04°/7.84°) in the case of MT-Unet_B vs. MT-Unet_D. Given the whole test dataset, APT was reduced by 4% and increased by 2% for MT-Unet_B vs. MT-Unet_D and MT-Unet_C vs. MT-Unet_A. Meanwhile, in the case of MT-Unet_C vs. MT-Unet_A, ∆AoPs were reduced for #3 (5.39° vs. 5.93°) and #1 (4.80° vs. 6.62°) but increased for #2 (11.85° vs. 3.10°). Improvements were obtained in the case of MT-Unet_B vs. MT-Unet_D. For the whole test dataset, the predicted AoP was closer to the labeled one, and the average ∆AoP is slightly reduced (Table 2).

#### 3.2.4. Effectiveness of the AFM Unit

The effects of the AFM module on the performance of MT-Unet were investigated through the following two sets of experiments: MT-Unet_D vs. MT-Unet_C and MT-Unet_B vs. MT-Unet_A. As is shown in Figure 5, though there are no big differences between methods without AFM and with AFM, Table 2 shows improvement in Acc, Dist_L, Dist_R, APT, and ∆AoP, but no noticeable improvement for Dice_ALL, Dice_PS, and Dice_FH on the whole dataset.

#### 3.2.5. Effectiveness of SLF

The SLF's effect on the performance of MT-Unet was investigated via a comparison with MT-Unet without SLF (i.e., MT-Unet_D). Figure 5(a) shows no discrete region inside and (or) outside the labeled area for the FH has been observed (e.g., #2) and segmented targets were closer to the annotated areas, especially for #1 and #3, in the case of MT-Unet_D. APT and ∆AoP were significantly improved for #1, #2, and #3. APT of MT-Unet_D vs. that of MT-Unet is 3.41° vs. 7.84° for #1, 3.49° vs. 5.64° for #2, and 2.80° vs. 6.87° for #3. And ∆AoP of MT-Unet_D vs. MT-Unet is 1.02° vs. 2.68° for #1, 5.07° vs. 10.25° for #2, and 3.73° vs. 6.66° for #3. Further statistical results are shown in Table 3.

### 3.3. Comparison of Our Method with the Existing Deep Learning Approach

To the best of our knowledge, there is currently only one study that is based on deep learning for automatic AoP measurement. From the function of the two methods (ours vs. Zhou et al. [33]), the approach of Zhou et al. does not have the function of selecting standard plane images and has not integrated the ECA module and SLF for accuracy improvement.

**Figure 6:**
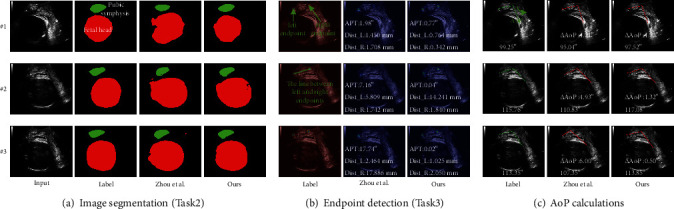
Our MT-Unet is compared to Zhou et al. [33]. (a) Compared to the predicted results of Zhou et al. (b) Endpoints obtained from ours and Zhou et al. are compared to the corresponding labels, respectively. (c) Calculations of AoP between Zhou et al. and ours. Note: Dist_L/Dist_R denotes the Euclidean distances between the predicted left/right endpoints and the true left/right endpoints. APT denotes the angle between the true line and the predicted line of two endpoints. ∆AoP denotes the absolute value of the AoP difference between the true AoP and the predicted one (complete and detailed results are provided in Appendix S4).

As shown in Figure 6(a), no discrete regions outside of the labeled area for the FH were observed in the case of ours vs. Zhou et al. Moreover, the area and shape of the segmented FH of our method are closer to the label. For endpoint detection (Figure 6(b)) and AoP calculation (Figure 6(c)), improvements have been found for both APT and ∆AoP in the three cases. APT of ours vs. Zhou et al. is 0.77° vs. 1.98° for #1, 0.04° vs. 7.16° for #2, and 0.02° vs. 17.74° for #3, and ∆AoP of ours vs. Zhou et al. is 1.73° vs. 4.21° for #1, 1.32° vs. 4.93° for #2, and 0.5° vs. 6.0° for #3. The results on the whole test dataset show that our methods yielded better performance than Zhou et al. on all metrics except Dice_PS (details can be found in Table 4).

### 3.4. Statistical Comparison of Our Method versus Clinical Manual Measurement

Evaluation of predicted AoP accuracy was carried out by making comparisons between maximum and minimum AoP in the test dataset including 289 images. On average, the absolute error in AoP between our method and clinical measurement is 3.898°.

**Figure 7:**
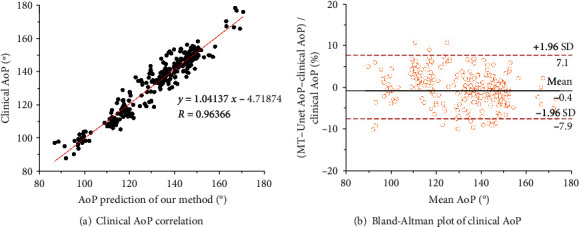
Clinical AoP vs. MT-Unet AoP. (a) Correlation plot of clinical and MT-Unet AoPs. (b) The Bland-Altman plot for comparison between automatic measurement and manual measurement results about AoP.

The linear regression plot in Figure 7(a) shows that the AoP estimates of both methods are linearly proportional and tightly clustered around the line of best fit *y* = 1.04137*x* − 4.71874 and the Pearson's correlation coefficient *R* = 0.964. In clinical studies, the standardized difference can determine a significant difference between the two results. There was 2.26° deviation between our method and clinical measurement, showing a subtle difference between the two methods.

Figure 7(b) is a Bland-Altman plot demonstrating the interchangeability of clinical measurement and our method for AoPs. The mean difference is -0.4. The magnitude of the AoP difference remained relatively constant for all mean AoP values, indicating that there is a systematic error instead of a proportional error between the two measurements. The limits of agreement are defined as Mean ± 1.96 SD, where SD is the standard deviation of the AoP differences. In this plot, 95% of the data points are within Mean ± 1.96 SD.

## 4. Discussions

Compared to the digital examination, the ultrasound examination is the more accurate and repeatable diagnosis of the FH position and the prediction of labor cessation. In clinical practice, doctors use their experience to first determine one standard plane image that includes the PS and the FH and then manually identify the three key points (i.e., two endpoints of the PS and the FH tangent point connected to the right endpoint of the PS) to calculate AoP based on the contour of the PS-FH. The application needs the experience in selecting standard section images from a large collection of TPU images. Furthermore, the identification of key points based on the contour of the PS-FH may introduce errors in the doctor's judgment. To overcome the disadvantages of manual measurement, we have presented a multitask deep learning model to achieve end-to-end fully automatic measurement of AoP. The main contributions of this work include the following: (1) to the best of our knowledge, this is the first study to achieve a fully automatic measurement of AoP. (2) We developed a multitask learning framework for standard plane image recognition, PS-FH segmentation, and key points identification. For the image classification task, it is committed to determining the standard plane, whereas image segmentation and position location tasks aim to obtain the contour of the PS-FH and endpoints of the PS, respectively. (3) We introduced attention mechanisms and an SLF in the MT-Unet for performance improvement. The AFM unit can capture cross-channel interaction to promote mutual learning between the segmentation branch and the location branch, while the ECA module can help avoid dimensionality reduction and capture cross-channel interaction. The convex shape prior loss can enhance robustness against noise and is of vital importance to the calculation of AoP, and (4) we adopted a two-stage training approach to make each branch of the network focus on its task.

Several steps have to be done to measure AoP: first, the standard plane images should be selected, then the contour of the PS-FH is detected, and the three key points of the detected contour are finally identified for AoP calculation. These three steps can be automatically conducted with our end-to-end MT-Unet, and thereby, our method is fully automatic. The other methods are based either fully or partly on standard plane images and include traditional methods and deep-learning methods. In the former category, Conversano et al. [32] proposed an algorithm that manually identifies the standard plane image first and adopts a pattern tracking algorithm for subsequent sessions to calculate AoP. Youssef et al. [54] reported an AoP measurement method based on commercial software; however, the technical characteristics of the software are not explained in detail [55]. In the deep learning-based category, Zhou et al. [33] applied an end-to-end deep learning method to measure AoP, but this approach is not fully automatic because it does not include image classification.

The high accuracy of our method is attributed to the use of ECA, AFM, and SLF. In the encoder of our network, the ECA module and Max-pooling are stacked together to capture the relation between adjacent channels and to compensate for the loss of information caused by downsampling. The performance of all branches is improved by the ECA module. In the decoders for Task2 and Task3, an AFM unit is used to capture cross-branch interaction between the segmentation branch (Task2) and the location branch (Task3). The segmented results include the areas of the PS and FH in Task2, while the predicted points are the two endpoints of the PS in Task3. Therefore, the AFM module can promote mutual learning (see Table 2). In addition, the accuracy of our method is further improved by SLF. The ideal shape of the FH appears elliptic in TPU images. We relax the elliptic-shape condition to the convex prior that enforces the segmentation result to be a convex polygon. The proposed SLF brings better results, which is helpful to perform ellipse fitting and find the tangent point. The AoP difference between predicted AoP and ground truth AoP is reduced by using the SLF (see Table 3). It should be mentioned that the accuracy (evaluated with ∆AoP) of the AoP calculation is higher than the existing deep learning method of Zhou et al. The fact that our results are more consistent with the experts' suggests that our method has the potential to be adopted in practice in the future.

Another cause of our outperforming multitask network is that different loss functions were applied for different tasks. This is because prior works showed that their best performance can only be achieved if the tuning is guided by task-specific loss functions and in turn by different evaluation metrics. For Task1, standard plane recognition is regarded as a binary classification problem, which is measured by accuracy. Considering that accuracy is a nonderivable equation, cross-entropy loss is chosen as the loss function. Dice loss is chosen as the loss function for Task2, similar to most prior medical image segmentation tasks. Additionally, we introduce SLF based on a convex polygon before modeling the area of PS and FH as a near-oval shape, which helps the calculation of AoP. For Task3, endpoint detection is an object localization problem, where Euclidean distance (e.g., MSE) is usually used to evaluate the deviation between the predicted object and the real object. MSE loss is chosen as the loss function accordingly. The training curves of MT-Unet are shown in Figure 1 of Appendix S5, which shows that our network is neither over- nor underfitting. In order to explore the effect of batch size on experimental results, we experimented with a higher batch size of 4 and 8. But we found that when batch size increased, the performance of Acc, Pre, and Spe dropped, so we still chose the batch size of 2 for our network (details are shown in Appendix S6).

Despite the better performance, the proposed approach still has pitfalls for future improvement: (1) different from other multitasking networks, a two-stage training strategy is adopted to obtain our MT-Unet due to the lack of labels for segmentation and location tasks in nonstandard plane images. (2) The effectiveness of this method on more data remains unknown. While random rotation and random scaling have been used for data augmentation during training to increase the number of limited data, the precision and generalization of this method remain to be investigated if given a much larger training dataset; (3) In this paper, the parameters of MT-Unet are 12.47 MB, and the computational complexity in GFLOPs of MT-Unet is 37.39, and we focus on accuracy improvement without considering computation complexity. In future research, we will work on the development of lightweight models without sacrificing accuracy; (4) inspired by the method of Conversano et al. [32], the accuracy of our method could be further improved by considering the relevance between images of a patient.

## 5. Conclusions

To the best of our knowledge, our method is an important step toward the fully automatic measurement of AoP. In the work, the proposed MT-Unet can perform the three tasks (i.e., image classification, image segmentation, and endpoint detection) for AoP calculation in a parallel manner. Our proposed neural network outperformed existing deep learning results.

## Figures and Tables

**Table 1:** Comparison of the results of standard plane recognition.

Network	Acc	Pre	Sen	Spe
Vgg16 [50]	90.87%	88.39%	86.19%	92.67%
Resnet50 [51]	90.28%	87.66%	85.87%	92.97%
ConvNet [52]	91.18%	86.76%	92.68%	90.14%
Swin_transformer [53]	92.18%	91.78%	90.54%	93.5%
Ours	92.26%	90.42%	86.57%	94.72%

**Table 2:** Comparison of the results of different methods.

Network	Acc (-)	Dice_ALL (-)	Dice_PS (-)	Dice_FH (-)	Dist_L (mm)	Dist_R (mm)	APT mean (STD) (°)	∆AoP mean (STD) (°)
Unet	97.1%	90.1%	80.0%	91.5%	6.948	11.414	12.496 (18.756)	6.646 (7.541)
MT-Unet_A	97.6%	91.2%	85.3%	91.9%	8.131	9.109	9.047 (13.263)	5.607 (5.725)
MT-Unet_B	98.2%	91.2%	85.2%	91.8%	9.105	8.198	6.960 (11.089)	6.696 (6.951)
MT-Unet_C	98.1%	91.9%	83.7%	92.8%	7.486	9.051	8.813 (6.640)	5.428 (5.022)
MT-Unet_D	98.4%	91.8%	85.8%	92.1%	6.638	5.586	6.614 (6.385)	5.053 (5.158)

**Table 3:** Comparison results on SLF.

Network	Acc (-)	Dice_ALL (-)	Dice_PS (-)	Dice_FH (-)	Dist_L (mm)	Dist_R (mm)	APT mean (STD) (°)	∆AoP mean (STD) (°)
MT-Unet_D	98.4%	91.8%	0.858%	92.1%	6.614	6.638	6.614 (6.385)	5.053 (5.158)
MT-Unet	98.9%	92.0%	84.8%	93.2%	6.303	5.789	6.500 (5.121)	3.898 (3.192)

Note: compared to MT-Unet, MT-Unet_D is without SLF.

**Table 4:** Comparison results of the proposed model and other deep learning methods.

Network	Acc (-)	Dice_ALL (-)	Dice_PS (-)	Dice_FH (-)	Dist_L (mm)	Dist_R (mm)	APT mean (STD) (°)	∆AoP mean (STD) (°)
Zhou et al. [33]	98.2%	91.2%	85.2%	91.8%	9.105	8.198	6.960 (11.089)	6.696 (6.951)
Ours	98.9%	92.0%	84.8%	93.2%	6.303	5.789	6.500 (5.121)	3.898 (3.192)

## Data Availability

The data used to support the findings of this study are available from the corresponding author upon request.

## Supplementary Materials

Supplementary Materials Appendix S1 is the details about how AoP is calculated, Appendix S2 is the detailed data of Figure 4, Appendix S3 is the detailed data of Figure 5, Appendix S4 is the detailed data of Figure 6, Appendix S5 is our training curves for MT-Unet, and Appendix S6 is about comparison results of different batch size.
