# Supplementary material for: Multitask Deep Neural Network for the Fully Automatic Measurement of the Angle of Progression
Source: Comput Math Methods Med. 2022 Sep 2;2022:5192338. doi: 10.1155/2022/5192338 (PMC9462992; doi:10.1155/2022/5192338)
Supplement: Supplementary Materials — Appendix S1 is the details about how AoP is calculated, Appendix S2 is the detailed data of Figure 4, Appendix S3 is the detailed data of Figure 5, Appendix S4 is the detailed data of Figure 6, Appendix S5 is our training curves for MT-Unet, and Appendix S6 is about comparison results of different batch size. [file 5192338.f1.docx]

**Appendix S1: the details about how AoP is calculated.**

### 2.2.1 Ellipse fitting of FH segmentation area

Considering the presence of noise in the image, we use median filtering to reduce noise in the segmentation result, and extract the contour of the FH. Then we use the following ellipse equation to fit the FH area.

$F\left( \boldsymbol{a},\boldsymbol{x} \right)=Ax^{2}+Bxy+Cy^{2}+Dx+Ey+F=0$ (1)

where ***a*** = [*A* *B C D* *E* *F*]*^T^* and ***x*** = [*x*^2^ *xy y*^2^ *x y* 1]*^T^*. *A* *B C D* *E* *F* are parameters, *x* and *y* are the horizontal and vertical coordinates. Our optimization goal is :

$\min{||\boldsymbol{a}^{T}\boldsymbol{x}||}^{2}$ (2)

We constrain the parameter vector ***a*** so that the conic that it represents is forced to be an ellipse to retain the efficiency of solution. The quadratic constraint is expressed in the matrix form ***a*** *^T^****Ca***=1 as

$\boldsymbol{a}^{T}\left[ \frac{\begin{aligned} 0 \\ 0 \\ 2 \end{aligned}}{\begin{aligned} 0 \\ 0 \\ 0 \end{aligned}} \right.\left. \frac{\begin{aligned} 0 \\ -1 \\ 0 \end{aligned}}{\begin{aligned} 0 \\ 0 \\ 0 \end{aligned}}\frac{\begin{aligned} 2 \\ 0 \\ 0 \end{aligned}}{\begin{aligned} 0 \\ 0 \\ 0 \end{aligned}}\frac{\begin{aligned} 0 \\ 0 \\ 0 \end{aligned}}{\begin{aligned} 0 \\ 0 \\ 0 \end{aligned}}\frac{\begin{aligned} 0 \\ 0 \\ 0 \end{aligned}}{\begin{aligned} 0 \\ 0 \\ 0 \end{aligned}}\frac{\begin{aligned} 0 \\ 0 \\ 0 \end{aligned}}{\begin{aligned} 0 \\ 0 \\ 0 \end{aligned}} \right]\boldsymbol{a}=1$ (3)

The result of equation (8) can be got, where *a* and *b* are the length of the long axis and the length of the short axis in the ellipse, (*x_0_*, *y_0_*) is the coordinates of the center point of the ellipse, $\theta$ is the rotation angle of the ellipse.

### 2.2.2 Determination of the tangent points

In order to determine the right tangent point connected to the right endpoint (*x_r_*, *y_r_*) of the PS, we establish a rectangular coordinate system (i.e., the long axis of the ellipse as the *x*’ axis and the short axis of the ellipse as the *y*’ axis). Thus, the ellipse equation of the FH is expressed as

$\frac{{x'}^{2}}{a^{2}}+\frac{{y'}^{2}}{b^{2}}=1$ (4)

Where the coordinates ($x_{r}^{'}$, $y_{r}^{'}$) of the right endpoint of the PS can be calculated as

$\left[ x_{r}^{'} y_{r}^{'} \right]=\left[ \frac{cos\theta}{sin\theta}\left. \frac{-sin\theta}{cos\theta} \right] \right.\left[ \left. \begin{aligned} x_{r}{-x}_{0} \\ y_{r}-y_{0} \end{aligned} \right] \right.$ (5)

We assume that the straight line that passes the right endpoint ($x_{r}^{'}$, $y_{r}^{'}$) of the PS is tangent to the ellipse curve.

$y^{'}=cx^{'}+d$ (6)

From the equations (12) and (14), we get two tangent points (i.e. ($x_{1}^{'},y_{1}^{'}$) and ($x_{2}^{'},y_{2}^{'}$)) and convert them into the original coordinate system. By comparing the abscissa values (i.e., *x_1_* and *x_2_*) of the two tangent points (i.e. (*x_1_*, *y_1_*) and (*x_2_*, *y_2_*)), we get the right tangent point (*x_t_*, *y_t_*).

$(x_{t}, y_{t})=\left\{ \begin{aligned} (x_{1}, y_{1}) \\ (x_{2}, y_{2}) \end{aligned} \right. \frac{if x_{1}\geq x_{2}}{otherwise}$ (7)

### 2.2.3 AoP calculation

Based on the three points (i.e., the right tangent point (*x_t_*, *y_t_*), the right endpoint (*x_r_*, *y_r_*) and the left endpoint (*x_l_*, *y_l_*) of the PS), the AoP is calculated as

$\vec{L_{1}}=[x_{r}-x_{l},y_{r}-y_{l}]$ (8)

$\vec{L_{2}}=[x_{t}-x_{r},y_{t}-y_{r}]$ (9)

$\mathrm{AoP}={cos}^{-1}\frac{\vec{L_{1}}\cdot\vec{L_{2}}}{\left| \vec{L_{1}} \right|\left| \vec{L_{2}} \right|}$ (10)

where the first line ($\vec{L_{1}}$) between (*x_r_*, *y_r_*) and (*x_l_*, *y_l_*) and the second line ($\vec{L_{2}}$) between (*x_r_*, *y_r_*) and (*x_t_*, *y_t_*) form an angle (i.e. AoP).

**Appendix S2: the detailed data of figure4.**

| Serial number | Dice_ALL  (-) | Dice_PS  (-) | Dice_FH  (-) | Dist_L  (mm) | Dist_R  (mm) | APT  (°) | $\Delta$AoP  (°) |
| --- | --- | --- | --- | --- | --- | --- | --- |
| #1 | 96.4% | 93.4% | 96.8% | 1.992 | 0.342 | 1.65 | 0.42(\|118.53-118.11\|) |
| #2 | 97.3% | 89.0% | 98.3% | 3.365 | 1.992 | 3.29 | 2.88(\|117.57-120.45\|) |
| #3 | 94.2% | 89.3% | 94.7% | 2.818 | 3.485 | 0.73 | 0.98(\|146.99-147.97\|) |

**Appendix S3: the detailed data of figure5.**

| Serial number | | Dice_ALL  (-) | Dice_PS  (-) | Dice_FH  (-) | Dist_L  (mm) | Dist_R  (mm) | APT  (°) | $\Delta$AoP  (°) |
| --- | --- | --- | --- | --- | --- | --- | --- | --- |
| #1 | Unet | 89.1% | 83.6% | 89.8% | 2.669 | 4.322 | 12.33 | 8.06(\|122.58-114.52\|) |
|  | MT-Unet_A | 91.9% | 88.7% | 92.2% | 3.094 | 11.281 | 10.95 | 6.62(\|121.14-114.52\|) |
|  | MT-Unet_B | 87.0% | 91.1% | 86.6% | 9.790 | 1.708 | 9.04 | 5.29(\|119.81-114.52\|) |
|  | MT-Unet_C | 94.6% | 90.7% | 95.0% | 2.292 | 3.485 | 8.89 | 4.80(\|119.32-114.52\|) |
|  | MT-Unet_D | 89.5% | 89.7% | 89.5% | 1.933 | 5.898 | 7.84 | 2.68(\|117.20-114.52\|) |
|  | MT-Unet | 98.0% | 93.9% | 98.4% | 0.342 | 0.764 | 3.41 | 1.02(\|115.54-114.52\|) |
| #2 | Unet | 90.5% | 79.3% | 91.7% | 9.660 | 4.322 | 4.78 | 11.14(\|151.53-140.39\|) |
|  | MT-Unet_A | 88.7% | 86.4% | 88.9% | 1.367 | 3.150 | 3.95 | 3.10(\|143.49-140.39\|) |
|  | MT-Unet_B | 85.1% | 86.3% | 85.0% | 14.420 | 3.598 | 4.41 | 11.29(\|151.68-140.39\|) |
|  | MT-Unet_C | 92.0% | 88.2% | 92.4% | 0.764 | 1.450 | 0.48 | 11.85(\|152.24-140.39\|) |
|  | MT-Unet_D | 91.8% | 88.3% | 92.2% | 0.483 | 1.367 | 5.64 | 10.25(150.64-140.39) |
|  | MT-Unet | 90.2% | 86.5% | 90.6% | 0.683 | 0.764 | 3.49 | 5.07(\|145.46-140.39\|) |
| #3 | Unet | 90.8% | 89.7% | 96.4% | 3.383 | 2.488 | 7.30 | 3.70(\|139.51-135.81\|) |
|  | MT-Unet_A | 94.5% | 90.6% | 94.9% | 2.755 | 3.383 | 5.54 | 5.93(\|129.88-135.81\|) |
|  | MT-Unet_B | 90.6% | 89.7% | 90.7% | 1.933 | 4.647 | 7.01 | 8.54(\|127.27-135.81\|) |
|  | MT-Unet_C | 95.0% | 90.9% | 95.4% | 3.150 | 3.820 | 6.47 | 5.39(\|130.42-135.81\|) |
|  | MT-Unet_D | 96.5% | 90.2% | 97.2% | 2.602 | 3.866 | 6.87 | 6.66(\|129.15-135.81\|) |
|  | MT-Unet | 92.3% | 89.5% | 92.6% | 2.669 | 3.696 | 2.80 | 3.73(\|132.08-135.81\|) |

**Appendix S4: the detailed data of figure6.**

| Serial number | | Dice_ALL  (-) | Dice_PS  (-) | Dice_FH  (-) | Dist_L  (mm) | Dist_R  (mm) | APT  (°) | $\Delta$AoP  (°) |
| --- | --- | --- | --- | --- | --- | --- | --- | --- |
| #1 | Zhou et al. | 95.5% | 88.8% | 96.7% | 1.450 | 1.708 | 1.98 | 4.21(\|95.04-99.25\|) |
|  | Ours | 93.8% | 84.6% | 95.3% | 0.764 | 0.342 | 0.77 | 1.73(\|97.52-99.25\|) |
| #2 | Zhou et al. | 89.2% | 87.4% | 89.4% | 5.809 | 1.742 | 7.16 | 4.93(\|110.83-115.76\|) |
|  | Ours | 87.9% | 86.4% | 88.1% | 1.424 | 1.840 | 0.04 | 1.32(\|117.08-115.76\|) |
| #3 | Zhou et al. | 92.4% | 88.5% | 92.9% | 2.464 | 17.886 | 17.74 | 6.00(\|107.35-113.35\|) |
|  | Ours | 90.1% | 88.0% | 90.3% | 1.025 | 2.050 | 0.02 | 0.50(\|113.85-113.35\|) |

**Appendix S5: Our training curves for MT-Unet.**


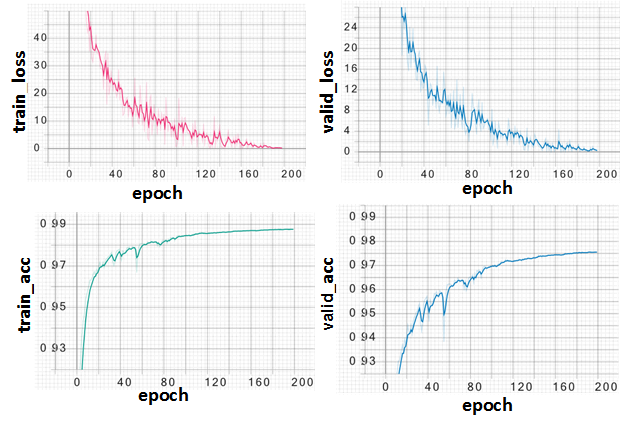


Fig. 1. Our training curves for MT-Unet.

**Appendix S6: Comparison results of different batch size.**

| batch size | Acc | Pre | Sen | Spe |
| --- | --- | --- | --- | --- |
| 8 | 91.37% | 88.95% | 89.66% | 91.32% |
| 4 | 90.98% | 87.67% | 90.68% | 90.66% |
| 2 (in our manuscript) | 92.26% | 90.42% | 86.57% | 94.72% |
